# Emergence of KPC-producing *Pseudomonas aeruginosa* in Spain: insights into an outbreak and resistance to the novel carbapenem/β-lactamase inhibitor combinations imipenem/relebactam and meropenem/vaborbactam

**DOI:** 10.1128/aac.01657-25

**Published:** 2026-03-27

**Authors:** Gloria Pérez-Rodríguez, Pablo Aja-Macaya, Lucía González-Pinto, Biel Taltavull, María Tarriño-León, María del Mar Gallardo-García, Tania Blanco-Martín, Salud Rodríguez-Pallares, Lucía Sánchez-Peña, Miriam Moscoso, Alejandro Beceiro, Antonio Oliver, Germán Bou, Jorge Arca-Suárez

**Affiliations:** 1 Servicio de Microbiología Clínica and Grupo de Investigación en Microbiología, Instituto de Investigación Biomédica de A Coruña (INIBIC), Complexo Hospitalario Universitario de A Coruña (CHUAC), SERGAS, Universidade da Coruña (UDC), A Coruña, Spain; 2 CIBER de Enfermedades Infecciosas (CIBERINFEC), Instituto de Salud Carlos III 637284 , Madrid, España; 3 Servicio de Microbiología, Servicio de Microbiología and Unidad de Investigación, Hospital Universitario Son Espases, Instituto de Investigación Sanitaria Illes Balears (IdISBa), Palma de Mallorca, Spain; 4 Servicio de Microbiología Clínica, Hospital Universitario Virgen de las Nieves, Granada, Spain; 5 Departamento de Fisioterapia, Medicina y Ciencias Biomédicas, Universidade da Coruña (UDC) 16737, A Coruña, Spain; University of Fribourg, Fribourg, Switzerland

**Keywords:** *Pseudomonas aeruginosa*, β-lactam resistance, β-lactamase, β-lactamase inhibitor, mutational mechanisms, KPC, carbapenemase, carbapenem, imipenem/relebactam, meropenem/vaborbactam

## Abstract

We report an outbreak of KPC-producing *Pseudomonas aeruginosa* in Spain, involving four patients and an environmental isolate recovered from a hospital sink. All isolates belonged to clone ST253 and exhibited difficult-to-treat resistance, including resistance to imipenem/relebactam and meropenem/vaborbactam. Genomic analysis confirmed a plasmid-encoded *bla*_KPC-2_ and OprD deficiency. Functional assays demonstrated synergy between KPC-2 production and porin loss on resistance to carbapenem/β-lactamase inhibitors. The findings highlight the clinical threat posed by KPC-producing *P. aeruginosa*.

## INTRODUCTION

*Pseudomonas aeruginosa* is classified by the World Health Organization as a high-priority pathogen due to its frequent involvement in severe nosocomial infections and its remarkable ability to develop resistance to all clinically available antipseudomonal agents, including carbapenems (1). Carbapenem resistance in *P. aeruginosa* is primarily driven by inactivating mutations in the outer membrane porin OprD, which typically confer resistance to imipenem and reduced susceptibility to meropenem (2). The accumulation of additional mutations affecting *ampC* expression and/or efflux pump activity is associated with high-level carbapenem resistance (3). Beyond mutational mechanisms, carbapenem resistance in *P. aeruginosa* is increasingly linked to the horizontal acquisition of carbapenemase-encoding genes, which is of a growing clinical concern (4). Infections caused by carbapenemase-producing strains are associated with significantly higher mortality rates than those caused by non-carbapenemase producers (5). Traditionally, metallo-β-lactamases (MBLs), such as VIM- and IMP-type enzymes, have been regarded as the predominant carbapenemases in this species. In addition, other less frequently reported acquired carbapenemases, including NDM-, AIM-, FIM-, and GES-type enzymes, have also been described in *P. aeruginosa* (6). However, in recent years, other enzymes that are more prevalent in Enterobacterales, such as KPC, are increasingly reported in clinical *P. aeruginosa* isolates worldwide (7). Alarmingly, according to the National Center for Biotechnology Information databases, *bla*_KPC_ ranks among the five most frequently detected horizontally acquired β-lactamase genes in available *P. aeruginosa* genomes (4). Circulation of KPC enzymes in *P. aeruginosa* appears to be particularly high in South America and Asia, whereas dissemination of these enzymes in Europe remains limited (8, 9). Nationwide surveys conducted in Spain in 2017 and 2022 did not detect any KPC-producing *P. aeruginosa* strains (10, 11), thus also suggesting a scarce dissemination in our country. KPC production in *P. aeruginosa* hinders the activity of most β-lactams, including ceftolozane/tazobactam. Nevertheless, the combinations ceftazidime/avibactam, imipenem/relebactam, and meropenem/vaborbactam generally retain some activity, owing to the potent inhibition of KPC enzymes by their respective β-lactamase inhibitors, and they may thus represent potentially useful options for treating KPC-producing *P. aeruginosa* infections (12).

Here, we sought to characterize a recent outbreak caused by KPC-producing *P. aeruginosa* in Spain, as well as to further examine the mechanisms of resistance limiting the activity of the newly developed carbapenem/β-lactamase inhibitors imipenem/relebactam and meropenem/vaborbactam.

Between January and April 2025, four clinical *P. aeruginosa* isolates were consecutively recovered from rectal surveillance (n = 1), sputum (n = 2), and urine (n = 1) samples obtained from four patients admitted to the Internal Medicine Unit of the Virgen de las Nieves University Hospital, Granada, Spain (Table 1; Table S1). Antimicrobial susceptibility testing was performed using the reference broth microdilution method. All determinations were carried out in duplicate and repeated in triplicate in cases where discordant results were observed between replicates. This analysis revealed a difficult-to-treat resistance (DTR) phenotype in all cases, characterized by resistance to ceftolozane/tazobactam, imipenem/relebactam, and meropenem/vaborbactam, with susceptibility only to ceftazidime/avibactam and cefiderocol retained. Notably, *P. aeruginosa* isolates 3-41184752 and 4-41184806 yielded approximately twofold increases in MIC values for most β-lactams. Preliminary detection of carbapenemases using in-house cloxacillin-inhibition tests, as well as multiplex lateral-flow immunochromatographic assays (O.K.N.V.I. RESIST-5, CORIS Bioconcept, Gembloux, Belgium), indicated the presence of a KPC-type carbapenemase in all cases. These findings were rather unexpected for two main reasons. First, KPC carbapenemases are extremely uncommon among *P. aeruginosa* isolates collected in Spain, and only very few cases have been described, always in association with MBLs. In a nationwide surveillance study conducted in 2016, two *P. aeruginosa* ST244 strains co-producing VIM-2 were detected (13). Four additional isolates producing KPC-2 or KPC-35 and co-harboring the MBL IMP-16 were later identified in patients of Peruvian and Spanish origin who received medical care in Barcelona (14). Second, the MIC values obtained for carbapenem/β-lactamase inhibitor combinations imipenem/relebactam and meropenem/vaborbactam, both of which usually display potent anti-KPC activity (15), were unexpectedly high, ranging from 8 to 32 mg/L and 64 to 256 mg/L, respectively.

**TABLE 1:** Case number, strain ID, antimicrobial susceptibility profile, and resistome of KPC-producing *P. aeruginosa* isolates

Case number-strain ID	MIC (mg/L)^*a*^*^,b^*																β-lactam acquired and mutational resistome^*c*^
	P/T	CAZ	C/A	C/T	ATM	A/A	FEP	F/T	F/Z	FDC	IMP	I/R	MEM	M/V	CIP	LEV	
	(R>16)	(R>8)	(R>8)	(R>4)	(R>16)	(R>16)	(R>8)	(R>8)	(R>8)	(R>2)	(R>4)	(R>2)	(R>8)	(R>8)	(R>0.5)	(R>2)	
1-41086362	256	64	2	32	>512	4	>512	8	8	0.25	256	8	512	64	>2	>4	*bla*_KPC-2_, *oprD* (D61fs), *mexZ* (V102_V105del), *pbpA* (A481T), *mpl* (D163G), *oprJ* (D68G; M69V), *mexD* (S685G), *mexC* (P383S; A384V)
2-41964617	256	64	2	32	>512	4	>512	8	8	0.25	256	8	512	64	>2	>4	*bla*_KPC-2_, *oprD* (D61fs), *mexZ* (V102_V105del), *pbpA* (A481T), *mpl* (D163G), *oprJ* (D68G; M69V), *mexD* (S685G), *mexC* (P383S; A384V)
3-41184752	512	256	8	64	>512	16	>512	32	16	0.125	256	32	>512	256	>2	>4	*bla*_KPC-2_, *oprD* (D61fs), *mexZ* (V102_V105del), *pbpA* (A481T), *mpl* (D163G), *oprJ* (D68G; M69V), *mexD* (S685G), *mexC* (P383S; A384V)
4-41184806	512	256	8	64	>512	16	>512	32	16	0.125	256	32	>512	512	>2	>4	*bla*_KPC-2_, *oprD* (D61fs), *mexZ* (V102_V105del), *pbpA* (A481T), *mpl* (D163G), *oprJ* (D68G; M69V), *mexD* (S685G), *mexC* (P383S; A384V)
Environmental isolate - 41166695	512	256	8	64	>512	16	>512	32	16	0.125	256	32	>512	512	>2	>4	*bla*_KPC-2_, *oprD* (D61fs), *mexZ* (V102_V105del), *pbpA* (A481T), *mpl* (D163G), *oprJ* (D68G; M69V), *mexD* (S685G), *mexC* (P383S; A384V)

^*a*^ P/T, piperacillin/tazobactam; CAZ, ceftazidime; C/A, ceftazidime-avibactam; C/T, ceftolozane-tazobactam; ATM, aztreonam; A/A, aztreonam-avibactam; FEP, cefepime; F/T, cefepime/taniborbactam; F/Z, cefepime/zidebactam; FDC, cefiderocol; IMP, imipenem; I/R, imipenem/relebactam; MEM, meropenem; M/V, meropenem/vaborbactam, CIP, ciprofloxacin; LEV, levofloxacin.

^*b*^ EUCAST v.15.0 breakpoints indicated.

^*c*^ del, deletion; fs, frameshift.

To gain further insight into the epidemiological features of this cluster, an epidemiological investigation was conducted in combination with whole-genome sequencing (WGS) using a hybrid assembly approach. Short- and long-read sequencing data generated with Illumina and PacBio technologies, respectively, were combined to obtain high-quality genome assemblies, as previously described by Rodríguez-Pallares *et al*. (16). The genomic information about these isolates is available on GenBank databases (PRJNA1330215). Epidemiological analysis revealed that all patients had received overlapping medical care in the same hospital room during consecutive admissions. A summary of the main clinical and demographic characteristics of the four patients is provided in Table S1. Based on these findings, and considering the known ability of *P. aeruginosa* to persist in wet environmental reservoirs, targeted sampling of wet surfaces was performed in the hospital room where the affected patients had been treated. Samples were cultured on CHROMID CARBA SMART Agar and CHROMID ESBL Agar (bioMérieux) and incubated for up to 48 h. Suspected colonies were identified as *P. aeruginosa*, and carbapenemase production was confirmed using the NG-Test CARBA-5 immunochromatographic assay. Only one sample, obtained from the sink faucet, yielded a positive result for KPC-producing *P. aeruginosa*. WGS revealed that all clinical and environmental isolates belonged to *P. aeruginosa* clone ST253, which is associated with a highly virulent profile and life-threatening infections due to production of the ExoU cytotoxin (17). Comparative core-genome phylogenomic analysis of this cluster with all *bla*_KPC_-positive *P. aeruginosa* genomes available in INSDC and RefSeq (n = 898 on 5 May 2025) showed close genomic relatedness to isolates from North America (Fig. S1). Pairwise whole-genome single-nucleotide polymorphism (SNP) analysis showed that all isolates involved in this association of cases differed by fewer than 14 SNPs (Table S2), indicating high genomic relatedness according to the threshold proposed by Schurch *et al*. (18) and confirming the outbreak. Interestingly, *P. aeruginosa* isolates 1-41086362 and 2-41964617, detected on January 10 and 18, were almost identical (SNP distance range: 2–5), whereas isolates 3-41184752 and 4-41184806 and the environmental isolate, identified 2 or more months later, formed another closely related cluster (SNP distance range: 2–6), with 7–12 SNP differences observed between the 2 clusters. These findings shed light on transmission between patients and the hospital environment and reinforce the role of hospital sinks and drains in the acquisition of DTR-*P. aeruginosa* (19). Importantly, following detection of KPC-producing *P. aeruginosa* in the sink faucet, patients were relocated, and the room was temporarily closed. The hospital maintenance service replaced the sink faucet and shower head and disinfected the corresponding plumbing sections. After these measurements were taken, follow-up environmental sampling yielded exclusively negative results. No additional cases of infection or colonization caused by KPC-producing *P. aeruginosa* were detected, and the outbreak was therefore considered to be terminated.

WGS was used to investigate the mechanisms of β-lactam resistance, with particular focus on those affecting imipenem/relebactam and meropenem/vaborbactam. Analysis of horizontally acquired resistance mechanisms confirmed the presence of *bla*_KPC-2_ located on a presumptively non-conjugative 38,545-bp IncP/IncU plasmid (Fig. S2). The gene is embedded within a Tn*3*-family composite transposon environment, flanked by multiple insertion sequences (IS*4*-like, IS*Kpn27*, IS*Apu2*, IS*Apu1*, and IS*630*) and associated with the Tn*1331* resolvase, suggesting a Tn*4401*-like transposon, described as being involved in *bla*_KPC_ mobilization (20). The prevalence of this plasmid was 6.12% (n = 55) in the aforementioned international *bla*_KPC_-positive *P. aeruginosa* dataset (Fig. S1). Additionally, a search for similar plasmids (35–45 Kbp and above 99% identity) in the Plasmid Database revealed a total of 62 entries, mostly involving species such as *Klebsiella pneumoniae* (n = 21, 33.9%) and *Citrobacter freundii* (n = 8, 12.9%) (21), thus confirming its broad dissemination in Enterobacterales and its potential mobilization to the *P. aeruginosa* background.

Analysis of the mutational resistome of the isolates investigated revealed an identical arrangement of β-lactam resistance mutations (Table 1). The most relevant changes putatively affecting β-lactam susceptibility included inactivation of OprD (D61fs), together with a 4-amino acid deletion affecting MexZ (ΔV102–V105; suggesting overexpression of the *mexXY* efflux operon). Interestingly, isolates 3-41184752 and 4-41184806 yielded markedly higher MIC values for almost all β-lactams than isolates 1-41086362 and 2-41964617 but without any apparent genomic differences affecting the β-lactam resistome. Relative expression analysis of *ampC*, efflux pump genes (*mexAB-oprM*, *mexXY*, *mexCD-oprJ*, and *mexEF-oprN*), and *bla*_KPC-2_ was assessed by quantitative real-time reverse transcription PCR as previously described by Cabral *et al*. (22). Gene expression levels were normalized to the transcription levels of the housekeeping gene *rpoS*. Relative expression was calculated using the comparative threshold cycle (2^–ΔΔCT^) method, assuming comparable amplification efficiencies for target and reference genes, and results were expressed as fold change relative to *P. aeruginosa* PAO1, which was used as the calibrator strain. It confirmed the expected *mexXY* overexpression (between 40- and 70-fold increase) in all isolates but did not identify substantial transcriptional differences in the other genes between *P. aeruginosa* isolates 1-41086362, 2-41964617, 3-41184752, and 4-41184806 that could explain the increased levels of β-lactam resistance observed in the latter (data not shown). Detailed analysis of differential mutations in other genes beyond those classically included as part of the β-lactam resistome revealed some changes and/or inactivating alterations in genes involved in metabolic functions: *cynR* (transcriptional regulator of the *cyn* operon, involved in cyanate metabolism), *mntH2* (manganese transporter belonging to the NRAMP family), *prpF* (2-methylaconitate isomerase), and *fdnG* (formate dehydrogenase-N subunit alpha). The contribution of such changes to β-lactam resistance should be experimentally addressed in specific studies.

Taken together, the clinical, genomic, and phenotypic data obtained in this study indicate that the unexpected resistance to newly developed carbapenem/β-lactamase inhibitor combinations with anti-KPC-2 activity may result from an interplay between KPC-2 production and impaired carbapenem uptake, a phenomenon previously described in resistant Enterobacterales (23, 24). To further elucidate this interplay, we assessed the impact of *bla*_KPC-2_ expression in both wild-type and OprD-deficient *P. aeruginosa* backgrounds. For this purpose, the *bla*_KPC-2_ gene was cloned into the pUCP24 plasmid as previously described (25), while the native IncP/IncU plasmid carrying *bla*_KPC-2_ was extracted from clinical isolates using the QIAGEN Plasmid Midi Kit (QIAGEN, Hilden, Germany) according to the manufacturer’s instructions. Both laboratory and clinical plasmids were then introduced in parallel into the naturally β-lactam-susceptible *P. aeruginosa* PAO1 strain and its imipenem-resistant, porin-deficient derivative *P. aeruginosa* PAOD1, which harbors a W65X premature stop codon (26), for comparative antimicrobial susceptibility testing. MIC analysis (Table 2) showed that KPC-2 production conferred resistance to all conventional β-lactams and to ceftolozane/tazobactam in *P. aeruginosa* PAO1, but ceftazidime/avibactam, imipenem/relebactam, and meropenem/vaborbactam remained susceptible (27). However, in *P. aeruginosa* PAOD1, *bla*_KPC-2_ expression further increased the resistance levels, in particular elevating MICs for imipenem/relebactam (from 0.25–0.5 to 4–8 mg/L) and meropenem/vaborbactam (from 4–8 to 64–128 mg/L). These results provide strong evidence of the synergistic effect between KPC production and decreased carbapenem uptake, confirming the combined role of both mechanisms in mediating resistance to novel carbapenem/β-lactamase inhibitor combinations in the *P. aeruginosa* background. Notably, meropenem/vaborbactam consistently exhibited higher MICs than imipenem/relebactam across different permeability backgrounds and genetic platforms. The lower activity of meropenem/vaborbactam in *P. aeruginosa* likely reflects the strong contribution of chromosomally encoded resistance mechanisms, including permeability loss and intrinsic efflux, together with a more limited functional contribution of vaborbactam in the *P. aeruginosa* background. In particular, meropenem activity in *P. aeruginosa* is known to be strongly influenced by these β-lactamase-independent mechanisms, while vaborbactam appears to have a more limited functional contribution in this background compared with relebactam. This may be related to reduced intracellular accumulation of vaborbactam, potentially due to limited penetration across the outer membrane and/or extrusion by intrinsic efflux systems such as MexAB-OprM, which have been shown to extrude boronate compounds (12, 25).

**TABLE 2:** Susceptibility profile of *P. aeruginosa* PAO1 and its OprD-deficient derivative *P. aeruginosa* PAOD1 complemented with recombinant and clinical plasmids encoding the *bla*_KPC-2_ gene

Strain	MIC (mg/L)^*a*^*^,b^*													
	P/T	CAZ	C/A	C/T	ATM	A/A	FEP	F/T	F/Z	FDC	IMP	I/R	MEM	M/V
	(R>16)	(R>8)	(R>8)	(R>4)	(R>16)	(R>16)	(R>8)	(R>8)	(R>8)	(R>2)	(R>4)	(R>2)	(R>8)	(R>8)
PAO1 (wild-type strain)	8	1	1	0.5	4	4	2	2	1	0.25	1	≤0.125	≤0.5	≤0.5
PAOD1 (spontaneous *oprD* null mutant (W65X) of PAO1)	8	1	1	0.5	4	4	2	2	1	0.125	8	0.5	2	2
PAO1 + pUCP-24 + KPC-2	128	256	4	64	>512	4	>512	2	4	0.5	16	0.25	64	4
PAO1 + IncP/IncU + KPC-2	256	128	4	32	>512	4	>512	4	8	0.5	32	0.5	128	8
PAOD1 + pUCP-24 + KPC-2	128	256	4	64	>512	4	>512	2	4	0.5	128	4	256	64
PAOD1 + IncP/IncU + KPC-2	256	128	4	32	>512	4	>512	4	8	0.5	256	8	512	128

^*a*^ P/T, piperacillin/tazobactam; CAZ, ceftazidime; C/A, ceftazidime/avibactam; C/T, ceftolozane/tazobactam; ATM, aztreonam; A/A, aztreonam/avibactam; FEP, cefepime; F/T, cefepime/taniborbactam; F/Z, cefepime/zidebactam; FDC, cefiderocol; IMP, imipenem; I/R, imipenem/relebactam; MEM, meropenem; M/V, meropenem/vaborbactam.

^*b*^ EUCAST v.15.0 breakpoints indicated.

In summary, this study describes an outbreak of KPC-producing *P. aeruginosa*, highlighting the emergence of a ST253 clone with high virulence and a DTR phenotype in Spain and Europe, where reports of KPC-producing *P. aeruginosa* remain scarce to date. Genomic investigation of isolates of clinical and environmental origin confirmed the close genetic relatedness, underscoring the potential of the clone to spread between patients and the hospital drainage system. Functional experiments with recombinant strains demonstrated that resistance to imipenem/relebactam and meropenem/vaborbactam, previously thought to remain active against KPC-producing Gram-negative strains, can arise from the synergistic effect of KPC-2 production and OprD inactivation in the *P. aeruginosa* background. These findings challenge the efficacy of carbapenem/β-lactamase inhibitors against KPC-producing *P. aeruginosa*, limiting last-line options to ceftazidime/avibactam and cefiderocol, against which resistance can also respectively emerge via amino acid changes affecting KPC structure or inactivating genetic alterations in iron-uptake systems (28, 29). Overall, the findings of this research underscore the remarkable adaptability of *P. aeruginosa* to acquire and combine resistance mechanisms and indicate the need to maintain active surveillance of this continuously evolving pathogen.

## SUPPLEMENTAL MATERIAL

Supplemental material aac.01657-25-s0001.docx Fig. S1 and S2; Tables S1 and S2.

ASM does not own the copyrights to Supplemental Material that may be linked to, or accessed through, an article. The authors have granted ASM a non-exclusive, world-wide license to publish the Supplemental Material files. Please contact the corresponding author directly for reuse.
