# Supplementary material for: Emergence of KPC-producing Pseudomonas aeruginosa in Spain: insights into an outbreak and resistance to the novel carbapenem/β-lactamase inhibitor combinations imipenem/relebactam and meropenem/vaborbactam
Source: Antimicrob Agents Chemother. 2026 Mar 27;70(5):e01657-25. doi: 10.1128/aac.01657-25 (PMC13148037; doi:10.1128/aac.01657-25)
Supplement: Supplemental material — Fig. S1 and S2; Tables S1 and S2. [file aac.01657-25-s0001.docx]

**Supplementary Material for**

**Emergence of KPC-producing *Pseudomonas aeruginosa* in Spain: insights into an outbreak and resistance to the novel carbapenem/β-lactamase inhibitor combinations imipenem/relebactam and meropenem/vaborbactam**

Gloria Pérez-Rodríguez^1^*, Pablo Aja-Macaya^1^*, Lucía González-Pinto^1,2^, Biel Taltavull^2,3^, María Tarriño-León^4^, María del Mar Gallardo-García^4^, Tania Blanco-Martín^1,2^, Salud Rodríguez-Pallares^1,2^, Lucía Sánchez-Peña^1^, Miriam Moscoso^1,2^, Alejandro Beceiro^1,2^, Antonio Oliver^2,3^, Germán Bou^1,2,5^, Jorge Arca-Suárez^1,2^

1. Servicio de Microbiología Clínica and Grupo de Investigación en Microbiología. Instituto de Investigación Biomédica de A Coruña (INIBIC), Complexo Hospitalario Universitario de A Coruña (CHUAC), SERGAS. Universidade da Coruña (UDC), A Coruña, Spain.
2. CIBER de Enfermedades Infecciosas (CIBERINFEC), Instituto de Salud Carlos III, Madrid, España.
3. Servicio de Microbiología, Servicio de Microbiología and Unidad de Investigación, Hospital Universitario Son Espases, Instituto de Investigación Sanitaria Illes Balears (IdISBa), Palma de Mallorca, Spain.
4. Servicio de Microbiología Clínica, Hospital Universitario Virgen de las Nieves, Granada, Spain.
5. Departamento de Fisioterapia, Medicina y Ciencias Biomédicas, Universidade da Coruña (UDC), A Coruña, Spain.

*Gloria Pérez-Rodríguez and Pablo Aja-Macaya contributed equally to this article. Author order was determined by consensus among all authors, taking into account their respective contributions to the study.

**Corresponding author:**

Dr. Jorge Arca-Suárez

e-mail: jorge.arca.suarez@sergas.es

Servicio de Microbiología Clínica & Grupo de Investigación en Microbiología. Instituto de Investigación Biomédica de A Coruña (INIBIC), Complexo Hospitalario Universitario de A Coruña (CHUAC), Sergas. Universidade da Coruña (UDC), As Xubias S/N, 3º Planta, 15006, A Coruña, Spain

CIBER de Enfermedades Infecciosas (CIBERINFEC), Instituto de Salud Carlos III, Madrid, España

Phone: 0034679082502

**Alternative corresponding author:**

Dr. Germán Bou

e-mail: german.bou.arevalo@sergas.es

Servicio de Microbiología Clínica & Grupo de Investigación en Microbiología. Instituto de Investigación Biomédica de A Coruña (INIBIC), Complexo Hospitalario Universitario de A Coruña (CHUAC), Sergas. Universidade da Coruña (UDC), As Xubias S/N, 3º Planta, 15006, A Coruña, Spain

CIBER de Enfermedades Infecciosas (CIBERINFEC), Instituto de Salud Carlos III, Madrid, España

Department of Physiotherapy, Medicine and Biomedical Sciences, University of A Coruña, A Coruña, Spain; Phone: 0034679082502

**Supplementary Material – Table of Contents**

- - - 1. Table S1 S4
      2. Figure S1 S5
      3. Table S2 S6
      4. Figure S2 S7

Table S1**.** Relevant demographic and clinical features of patients and samples testing positive for the KPC-producing *P. aeruginosa*

|  | **Patient data** | | | | | | | | **Sample data** | | | |
| --- | --- | --- | --- | --- | --- | --- | --- | --- | --- | --- | --- | --- |
| **Case number** | **Age** | **Sex** | **Date of admission** | **Reason for admission** | **Infection / colonization** | **Previous antibiotherapy*^a^*** | **Other relevant clinical features*^b^*** | **30-day evolution** | | **Date of first positive sample** | **Days from admission until first positive sample** | **Type of sample** |
| 1-41086362 | 76 | M | 27/12/2024 | Urinary sepsis | Colonization | CTA, LEV, LIN, MEM, P/T | T2DM, COPD | Discharge home | | 18/01/2025 | 22 days | Rectal |
| 2-41964617 | 81 | M | 27/12/2024 | Pneumonia | Infection | AMC, CIP, ERT, LIN, MET | T2DM | Exitus | | 10/01/2025 | 14 days | Sputum |
| 3-41184752 | 92 | M | 01/02/2025 | Acute cholecystitis | Infection / colonization | AMC, CTR, C/A, DAP, LIN, MEM, TED | T2DM, COPD, abscessed infection (*S. aureus*), HAP (*E. coli*), UTI (*E. faecium*) | Discharge home | | 17/03/2025 | 44 days | Sputum |
| 4-41184806 | 89 | M | 29/03/2025 | Lung neoplasm + renal failure | Infection | AMC, C/A, ERT | COPD | Exitus | | 11/04/2025 | 19 days | Urine |

^a^CTA, cefotaxime; LEV, levofloxacin; LIN, linezolid; MEM, meropenem; P/T, piperacillin/tazobactam; AMC, amoxicillin/clavulanate; CIP, ciprofloxacin; ERT, ertapenem; MET, metronidazole; CTR, ceftriaxone; C/A, ceftazidime/avibactam; DAP, daptomycin; TED, tedizolid.

^b^T2DM, Type 2 Diabetes Mellitus; COPD, Chronic Obstructive Pulmonary Disease; HAP, Hospital-Acquired Pneumonia; UTI, Urinary Tract Infection.


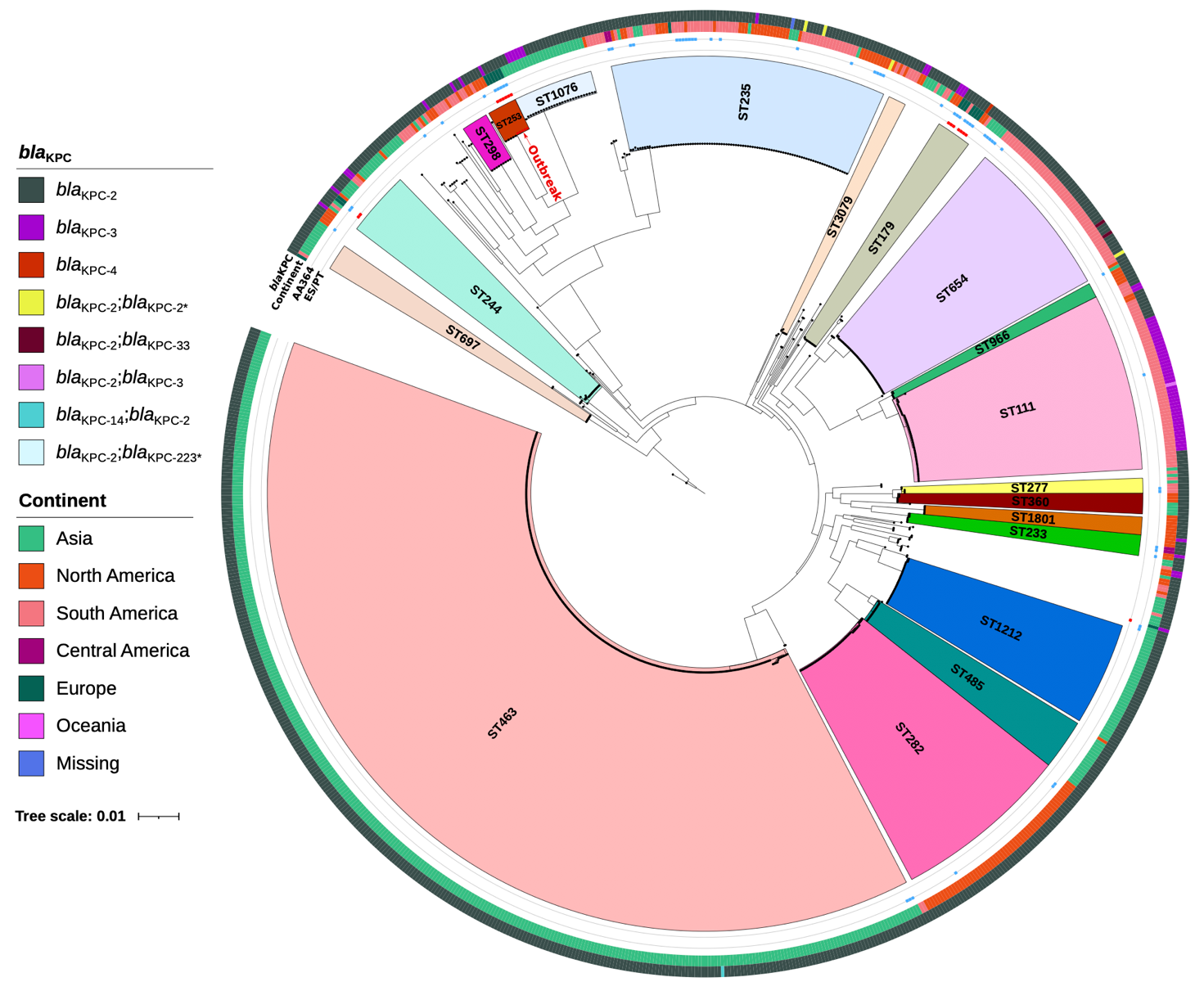


Figure S1**.** Core genome phylogenomic tree showing all KPC-producing *P. aeruginosa* assemblies available in INSDC and RefSeq up to 2025-05-05 (n=898), with *P. aeruginosa* PAO1 (GCF_000006765.1) as the reference strain. Presence of the plasmid identified in this article (named AA364 by MOB-suite) is indicated by blue dots around the tree. Similarly, red dots around the tree denote assemblies from Spain or Portugal (ES/PT). Leaves corresponding to the outbreak identified in this study are also shown in red. The most frequent Multi-Locus Sequence Types (MLSTs) are highlighted with coloured areas.

Table S2**.** Number of mutations (including single nucleotide substitutions, insertions, and deletions) identified in pairwise whole-genome comparisons between the five isolates. Each cell represents the total number of variants detected when using the genome of the isolate indicated in the row as the reference for variant calling against the isolate indicated in the column. Variant calling was performed using Illumina sequencing data

|  | 1-41086362 | 2-41964617 | 3-41184752 | 4-41184806 | Environmental isolate |
| --- | --- | --- | --- | --- | --- |
| 1-41086362 | 1 | 2 | 9 | 8 | 10 |
| 2-41964617 | 5 | 1 | 12 | 11 | 12 |
| 3-41184752 | 8 | 8 | 1 | 2 | 3 |
| 4-41184806 | 14 | 14 | 5 | 4 | 6 |
| Environmental isolate | 7 | 7 | 2 | 2 | 2 |


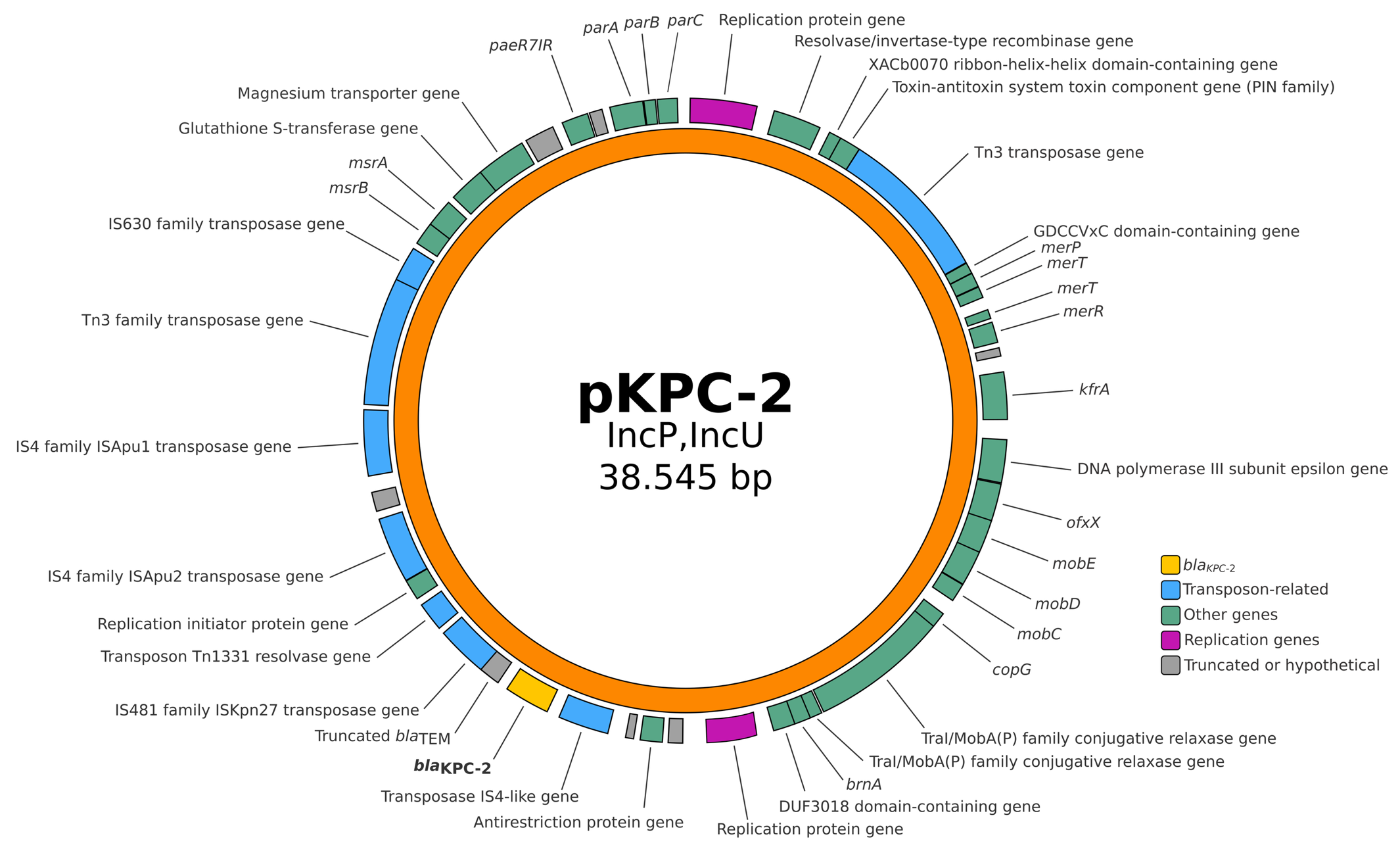


Figure S2**.** Annotated representation of the gene products encoded by the *bla*_KPC-2_-carrying Incp-IncU plasmid, pKPC-2 (named AA364 by MOB-suite), identified in this study.
